# Multilocus definition of MHC haplotypes in pedigreed cynomolgus macaques (*Macaca fascicularis*)

**DOI:** 10.1007/s00251-012-0632-2

**Published:** 2012-07-08

**Authors:** Nel Otting, Nanine de Groot, Annemiek J. M. de Vos-Rouweler, Annet Louwerse, Gaby G. M. Doxiadis, Ronald E. Bontrop

**Affiliations:** Department of Comparative Genetics and Refinement, Biomedical Primate Research Centre, Lange Kleiweg 161, 2288GJ Rijswijk, The Netherlands

**Keywords:** Nonhuman primates, MHC, Cynomolgus, Long-tailed, Crab-eating macaques, Haplotype

## Abstract

Cynomolgus macaques (*Macaca fascicularis*) are used widely in biomedical research, and the genetics of their MHC (*Mhc-Mafa*) has become the focus of considerable attention in recent years. The cohort of Indonesian pedigreed macaques that we present here was typed for *Mafa-A*, *-B*, and *-DR*, by sequencing, as described in earlier studies. Additionally, the *DRB* region of these animals was characterised by microsatellite analyses. In this study, full-length sequencing of *Mafa-DPA/B* and *-DQA/B* in these animals was performed. A total of 75 different alleles were observed; 22 of which have not previously been reported, plus 18 extended exon 2 alleles that were already known. Furthermore, two microsatellites, D6S2854 and D6S2859, were used to characterise the complex *Mafa-A* region. Sequencing and segregation analyses revealed that the length patterns of these microsatellites are unique for each *Mafa-A* haplotype. In this work, we present a pedigreed colony of approximately 120 cynomolgus macaques; all of which are typed for the most significant polymorphic MHC class I and class II markers. Offspring of these pedigreed animals are easily characterised for their MHC by microsatellite analyses on the *Mafa-A* and *-DRB* regions, which makes the cumbersome sequencing analyses redundant.

## Introduction

Nonhuman primates are often used in biomedical research as animal models for a variety of human diseases. Traditionally, the rhesus macaque (*Macaca mulatta*) has been the preferred species. In recent years however, cynomolgus macaques (*Macaca fascicularis*), also known as long-tailed or crab-eating macaques, have emerged in a number of studies. Whereas the rhesus macaques originate in Southern Asia and China, the cynomolgus macaques are found in Southeast Asia, including the Filipinos, and on the East African Island of Mauritius. They are used in research on infectious diseases such as AIDS, tuberculosis, and dengue (Cafaro et al. 2010; Chen et al. 2007; Langermans et al. 2001; Mee et al. 2009). Moreover, they are applied as models for Alzheimer’s and Parkinson’s disease, and in transplantation studies (Emborg 2007; Haustein et al. 2010; Wang et al. 2007).

Because of the animals’ role as models for immunological disorders, the Major histocompatibility complex (MHC) of the cynomolgus macaque (*MhcMafa*) is the subject of investigations. The highly polymorphic genes of the MHC encode cell-surface proteins, of which the most relevant are the classical class I and class II molecules. These proteins play a critical role in immunological responses to pathogens and hence may have a profound impact on disease susceptibility and organ transplantation (Bontrop 2006; Parham and Ohta 1996). In addition, the genetics of the killer cell Ig-like receptor (KIR) polymorphisms are currently being studied in these animals (Bimber et al. 2008).

Molecular genetic analyses of the class I genes in different populations of cynomolgus monkeys have led to hundreds of full-length *Mafa-A* and *-B* alleles (Aarnink et al. 2011; Budde et al. 2010; Campbell et al. 2009; Kita et al. 2009; Krebs et al. 2005; Lawrence et al. 2012; Ling et al. 2012; Mitchell et al. 2011; Otting et al. 2007, 2009; Pendley et al. 2008; Saito et al. 2011; Uda et al. 2004; Wiseman et al. 2009; Zhuo et al. 2011); all of which have been assembled in the IPD-MHC database (de Groot et al. 2012; Robinson et al. 2003). These studies have shown that most alleles are specific for the geographic origins of the animals, a phenomenon that is also observed in rhesus macaques (Otting et al. 2005, 2008). This observation dictates that each cohort of animals that is used in biomedical research be investigated for its repertoire of MHC alleles. A relatively restricted MHC diversity is present in macaques of Mauritian origin, which makes them useful in studies that require MHC-identical animals (Budde et al. 2010; Cafaro et al. 2010; Krebs et al. 2005; Mee et al. 2009). The class I alleles observed in this homogenous population are shared with those of animals originating in Indonesia, which confirms the founder effect as regards animals that originated in this archipelago.

The most intensively investigated class II genes are *DRA* and *-B*, *DQA* and *-B*, and *DPA* and *-B* (Bontrop et al. 1999; Creager et al. 2011; de Groot et al. 2004, 2008; Doxiadis et al. 2007, 2006; Hashiba et al. 1993; Leuchte et al. 2004; Ling et al. 2011; O’Connor et al. 2007; Otting et al. 2002; Sano et al. 2006). The proteins are expressed as heterodimers, consisting of alpha and beta chains that are encoded by the *A* and *B* genes. In recent years, sequencing analyses of class II genes have been performed mostly on exon 2, since these polymorphic exons encode the peptide binding domains of the heterodimers. However, the alignments of full-length class II sequences in the IPD-MHC database reveal alleles that share exon 2 but that differ in other exons (Creager et al. 2011). In comparison to class I alleles in the cynomolgus macaques, the class II alleles appear to be less specific for the geographic origins of the animals.

The Biomedical Primate Research Centre (BPRC) houses a population of cynomolgus macaques that has been pedigreed for up to seven generations. Most animals were sequenced for the class I *A* and *B* (Otting et al. 2007, 2009) and for the class II *DR* regions (Doxiadis et al. 2006), and these analyses were supplemented with microsatellite analyses for the *DRB* region (de Groot et al. 2008; Doxiadis et al. 2010). From a subset of these animals, *DQB* sequencing of exon 2 has been performed (Doxiadis et al. 2006; Otting et al. 2002). With the aim of completing the map of haplotypes, we performed sequencing analyses of *DPA*, *DPB*, *DQA*, and *DQB* genes. Since polymorphism of class II genes may be present in exons other than exon 2, we performed full-length sequencing. Furthermore, with the intention of avoiding future time-consuming sequencing analyses on newborn animals in this colony, the *Mafa-A* region was characterised by microsatellite analyses (Doxiadis et al. 2011a; Penedo et al. 2005).

## Materials and methods

### Animals and cell lines

The BPRC houses a self-sustaining colony of about 120 cynomolgus macaques. Before being transferred to the BPRC facilities, the animals were pedigreed in 12 matrilines for up to seven generations at the University of Utrecht. Beginning with ten animals in the late 1960s, the colony was gradually extended by a few males and three females in the 1970s, nine females and their offspring in the 1980s, and three males in the 1990s, all from different institutions. The estimated number of founder animals is therefore at least 30. The animals originated in the Indonesian islands and continental Malaysia, as defined by mitochondrial (12S RNA) analyses (Doxiadis et al. 2006).

Lymphoblastoid B-cell-lines and genomic DNA are available from most animals in the colony. In addition, samples are present from related animals that are no longer available. The animals were typed earlier for *Mafa-A*, *-B*, and *-DR* (Doxiadis et al. 2011b; Otting et al. 2007, 2009), and the combination of these data resulted in 30 different haplotypes. Since mitochondrial DNA and MHC haplotypes are inherited independently and the founder animals of the colony have died, it is not possible to distinguish unambiguously between Indonesian and Malaysian MHC haplotypes. For these reasons, all haplotypes are considered as Indonesian. To sequence the full-length *DP* and *DQ* sequences, we chose at least three animals, preferably from different matrilines, for each haplotype in the colony. The genomic DNA samples of all animals were included for *Mafa-A* microsatellite or short tandem repeat (STR) analyses.

### cDNA, cloning, and sequencing

RNA was isolated from B-lymphocytes (Rneasy kit, Qiagen) and subjected to One-Step RT-PCR, as recommended by the supplier (Promega). The primers used for the amplification of *DPA*, *DPB*, *DQA*, and *DQB* transcripts were copied from a study on Mauritian cynomolgus macaques (O’Connor et al. 2007). Additional primers are DPA-F-inex: GACAGAATGTTCSAGACCAG, DPA-R-inex: CGTTGTCTCAGGSATCTGGAT, DPB-F-inex: GGCRTTACTGATGGTRCTGC, DPB-R-inex: CCTCCTGTGCATGAAGATGCCC, DQA-F-inex: CAAAGCTCTGWTGCTGGSGG, and DQA-R-inex: GCCCTTGGTGTCTGGARGC. The final elongation step was extended to 7 min to generate a 3′dA overhang. The RT-PCR products were cloned using the PCR cloning kit (Qiagen). After transformation, 16 colonies were selected for plasmid isolation. Sequencing reactions were performed using the BigDye terminator cycle sequencing kit, and samples were run on an automated capillary sequencing system (Applied Biosystems Genetic Analyzer 3130).

Sequences were analyzed using MacVector^™^ version 10.6.0 (Oxford Molecular Group), followed by manual adjustments. New alleles, represented by three identical clones, present in at least two different animals or RT-PCR samples, were submitted to the EMBL-EBI and, for official designations, to the nonhuman primate section of the IMGT/MHC Immuno Polymorphism Database (de Groot et al. 2012; Robinson et al. 2003).

### *Mafa-A* microsatellite typing

The polymerase chain reaction (PCR) amplifications, with primers for the microsatellites D6S2854, a (TAAA)n repeat, and D6S2859, a mixed dinucleotide (TA)×(CA)y repeat, were performed under the same conditions as described previously for the rhesus macaques at BPRC (Doxiadis et al. 2011a). Briefly, both PCR reactions on genomic DNA samples were multiplexed in a 25-μl mixture. The amplified DNA fragments were mixed with size standards and separated on an automated capillary electrophoresis system (Applied Biosystems Genetic Analyzer 3130). Fragment-length analysis was performed using the Genemapper software (Applied Biosystems).

## Results

### Novel alleles

#### *Mafa-DPA1/DPB1*

In the panel of cynomolgus macaques, 17 different *Mafa-DPA1* sequences were detected. All *DPA1* alleles were amplified with the MHCII-DPA-F/R primer pair (O’Connor et al. 2007). For animals that showed only one allele in the RT-PCR, an extra amplification was performed with the DPA-inex (in exon) primers. These inex-primers were designed so that they anneal to the outermost exons of all known alleles in the IPD-MHC database. In some instances, the second allele was found, indicating that certain alleles may be amplified abundantly, at the expense of the one present on the other haplotype. These minor alleles were sufficiently present in other animals, however. Eight alleles had not been reported previously, whereas another one extended a known *DPA1* exon 2 sequence. These alleles were submitted to the EMBL/EBI and, for official designations, to the IPD-MHC database. The sequences are listed in Table 1, including the accession numbers, reference animals, and the geographic origin of the animals.

**Table 1:** The DP and DQ alleles detected in this study and the EMBL accession numbers. For the novel and the extended alleles, a reference animal is included. The origins are indicated by *ind*, *mal*, *mau*, and *vie* that stand for Indonesian, Malaysian, Mauritian, and Vietnamese, respectively. The PCR-primer pairs that amplified the detected alleles are listed in the last column

*MhcMafa*	Accession		Animal	Origin	Primer pairs
DPA1*02:01	HE573230	Extension		Ind	MHCHII-DPA-F/R
DPA1*02:02	EF208806			Ind, mau	MHCHII-DPA-F/R
DPA1*02:03	EF208807, HM579973			Ind, mau	MHCHII-DPA-F/R
DPA1*02:06:02	FR719027	Novel	Mesa	Ind	MHCHII-DPA-F/R
DPA1*02:08:01	HM580029			Ind, vie	MHCHII-DPA-F/R
DPA1*02:13:02	FR719025	Novel	Tremaa	Ind	MHCHII-DPA-F/R
DPA1*02:16	HM579972, FR719028		Amaretto	Ind	MHCHII-DPA-F/R
DPA1*02:19	FR719022	Novel	Donna	Ind	MHCHII-DPA-F/R
DPA1*02:20	FR719023	Novel	Voodoo	Ind	MHCHII-DPA-F/R
DPA1*02:21	FR719024	Novel	Caya	Ind	MHCHII-DPA-F/R
DPA1*02:22	FR719029	Novel	Kaa	Ind	MHCHII-DPA-F/R
DPA1*02:23	HE573229	Novel	Cornea	Ind	MHCHII-DPA-F/R
DPA1*04:01	AF208808			Ind, mau	MHCHII-DPA-F/R
DPA1*04:02	HM579969			Ind	MHCHII-DPA-F/R
DPA1*07:02	EF208810			Ind, mau	MHCHII-DPA-F/R
DPA1*07:04	HM579964, FR719026		Falcao	Ind	MHCHII-DPA-F/R
DPA1*10:01	FR719021	Novel	Varoa	Ind	MHCHII-DPA-F/R
DPB1*01:07	FR719032	Novel	Mesa	Ind	MHCHII-DPB-F1/R1 and R2
DPB1*02:02	HM580039			Ind, vie	MHCHII-DPB-F1/R1
DPB1*02:05	FR719033	Novel	Mojo	Ind	MHCHII-DPB-F1/R1 and R2
DPB1*03:01	HM580037			Ind, vie	MHCHII-DPB-F1/R2
DPB1*03:03	AM086069			Ind, mau	MHCHII-DPB-F1/R1 and R2
DPB1*03:04	HM579981			Ind, vie	MHCHII-DPB-F1/R1
DPB1*04:01	EF208811			Ind, mau	MHCHII-DPB-F1/R1 and R2
DPB1*06:01	HM580038			Ind, vie	MHCHII-DPB-F1/R1 and R2
DPB1*06:03	HE573231	Extension	Alfa	Ind	MHCHII-DPB-F1/R2
DPB1*06:08	HM579983			Ind	MHCHII-DPB-F1/R1 and R2
DPB1*07:01	HM580035			Ind	MHCHII-DPB-F1/R2
DPB1*09:02	EF208813, HM579980			Ind, mau	MHCHII-DPB-F1/R1
DPB1*10:01	HM579982			Ind	MHCHII-DPB-F1/R1
DPB1*15:02	HM579977			Ind	MHCHII-DPB-F1/R1 and R2
DPB1*15:03	AM943636			Ind	MHCHII-DPB-F1/R1 and R2
DPB1*15:05	FR719031	Extension	Voodoo	Ind	MHCHII-DPB-F1/R1 and R2
DPB1*15:08	HE753232	Novel	Dobo	Ind	MHCHII-DPB-F1/R1 and R2
DPB1*18:01	HE611949	Extension	Roza	Ind	DPB-F/R-inex only
DPB1*19:03	EF208814			Ind, mau	DPB-F/R-inex only
DPB1*20:01	EF208815			Ind, mau	DPB-F/R-inex only
DPB1*21:01	HE611948	Novel	Geisha	Ind	DPB-F/R-inex only
DQA1*01:02	FR719042	Extension	Pachuca	Ind	MHCHII-DQA-F2/R2
DQA1*01:15	HE573234	Extension	Joshua	Ind	MHCHII-DQA-F2/R2
DQA1*01:06	EF208817			Ind, mau	MHCHII-DQA-F2/R2
DQA1*01:07	EF208818, HM579989			Ind, mau	MHCHII-DQA-F2/R2
DQA1*01:08:02	FR719041	Novel	Sjerpa	Ind	MHCHII-DQA-F2/R2
DQA1*01:13	FR719037	Novel	Bamboo	Ind	MHCHII-DQA-F2/R2
DQA1*01:14	FR719038	Novel	Kaa	Ind	MHCHII-DQA-F2/R2
DQA1*05:02	FR719043	Extension	Tjatjatja	Ind	MHCHII-DQA-F2/R2
DQA1*05:03:01	EF208819, FR719035		Mesa	Ind, mau	MHCHII-DQA-F2/R2 and F1/R1
DQA1*05:03:02	HM579988			Ind, vie	MHCHII-DQA-F2/R2
DQA1*05:04	FR719045	Extension	Suraya	Ind	MHCHII-DQA-F2/R2
DQA1*05:07	FR719034	Novel	Caya	Ind	MHCHII-DQA-F2/R2
DQA1*05:08	FR719039	Novel	Domino	Ind	MHCHII-DQA-F2/R2 and F1/R1
DQA1*05:09	FR719040	Novel	Varoa	Ind	MHCHII-DQA-F2/R2
DQA1*23:01	FR719044	Extension	Mojo	Ind	MHCHII-DQA-F2/R2 and F1/R1
DQA1*24:02:01	AM943643			Ind	DQA-F/R-inex only
DQA1*24:02:03	HE611950	Novel	Geisha	Ind	MHCHII-DQA-F1/R1
DQA1*24:03	EF208820, HE573233			Ind	DQA-F/R-inex only
DQA1*24:06	HE573235	Novel	Xueso	Ind	MHCHII-DQA-F2/R2
DQA1*26:01:02	FR719036	Novel		Ind	MHCHII-DQA-F2/R2 and F1/R1
DQB1*06:01:02	EF442017			Ind, mau	MHCHII-DQB-F2/R2
DQB1*06:07:01	HM579998			Ind	MHCHII-DQB-F2/R2
DQB1*06:08	EF208822			Ind, mau	MHCHII-DQB-F2/R2
DQB1*06:11	EF208823, HM153008			Ind, mau	MHCHII-DQB-F2/R2
DQB1*06:15	FR719054	Extension	Colorado	Ind	MHCHII-DQB-F2/R2
DQB1*06:17	FR719048	Extension	Bamboo	Ind	MHCHII-DQB-F2/R2
DQB1*06:23	HE573237	Extension	Kaa	Ind	MHCHII-DQB-F2/R2
DQB1*16:01	FR719046	Extension	Mesa	Ind	MHCHII-DQB-F2/R2
DQB1*17:02:01	FR719051			Ind, vie	MHCHII-DQB-F2/R2
DQB1*17:02:02	FR719049	Novel		Ind	MHCHII-DQB-F2/R2
DQB1*17:04	FR719053	Extension		Ind	MHCHII-DQB-F2/R2
DQB1*17:05	FR719052	Extension		Ind	MHCHII-DQB-F2/R2
DQB1*17:06:02	FR719050	Extension		Ind	MHCHII-DQB-F2/R2
DQB1*18:01:01	EF208825			Ind, mau	MHCHII-DQB-F2/R2
DQB1*18:04	FR719047	Extension		Ind	MHCHII-DQB-F2/R2
DQB1*18:06	HE573238	Extension		Ind	MHCHII-DQB-F2/R2
DQB1*18:08	EZ933325, HE573236			Ind, mal	MHCHII-DQB-F2/R2

The *DPB1* amplification was initiated with the MHCII-DPB-F1/R1 primer pair (O’Connor et al. 2007), though for those samples with only one allele, we performed second RT-PCRs with another reverse primer: MHCII-DPB-R2. In the event that this did not yield a second allele, the samples were subjected to RT-PCR with DPB-inex primers that anneal to the exons in all known *Mafa-DPB* sequences. Using these primers, four alleles were found, which were not detected with the full-length primers in any of the animals. A total of 21 *Mafa-DPB1* sequences were observed within the cohort (Table 1). Four alleles were submitted as novel sequences, whereas three others extended known exon 2 sequences. Two of the latter are not full-length class II sequences because they were detected using the DPB-inex primers.

#### *Mafa-DQA/DQB*

Twenty *Mafa-DQA* alleles were present in the cohort, of which nine were novel, and five extended known exon 2 sequences. The analyses were initiated using the DQA-F2/R2 primers and extended with DQA-F1/R1 and DQA-inex primers in the event that alleles were not amplified. Finally, 17 *DQB* alleles were found with DQB-F2/R2; nine of which extended known exon 2 sequences. One allele was submitted as a novel sequence.

Among the 75 sequences detected, 16 are shared with alleles of Mauritian origin (O’Connor et al. 2007). This is not surprising, since the founders of this colony are assumed to have originated in the Indonesian archipelago. Seven alleles were also detected in animals of Vietnamese descent. This confirms earlier findings of shared class II alleles in animals of Vietnamese and Indonesian descent (Creager et al. 2011). One allele was seen earlier in a Malaysian cohort (Aarnink et al. 2011).

### *Mafa-A* microsatellite typing

The microsatellites D6S2854 and D6S2859 have been used earlier to type the different groups of rhesus macaques housed at BPRC. These analyses have shown that both markers are polymorphic in length and that the typing results are reproducible. Genotyping resulted in patterns of varying length that, based on segregation analyses, are associated with the *Mamu-A* haplotypes. Since all of these haplotypes and associated microsatellite patterns are known within the rhesus breeding groups, this technique is applied as a fast typing method for screening the animals’ offspring. The cynomolgus and rhesus macaques share the organisation of the MHC class I region (Otting et al. 2007; Watanabe et al. 2007), and the same microsatellite method was expected to be suitable for genotyping the cohort of cynomolgus macaques*.* Indeed, the length patterns of the D6S2854 and D6S2859 PCR fragments matched with the 30 *Mafa-A/-B/-DRB* haplotypes in the cohort.

### *MhcMafa* haplotypes

Based on earlier studies on the class I *A* and *B* as well as on the class II *DR* regions, it was possible to define 30 haplotypes within the colony of animals. In this study, we were able to append the *DP* and *DQ* full-length alleles in addition to the D6S2854 and D6S2859 microsatellite length patterns. An overview of the haplotypes is provided in Table 2. The gene regions are listed according to their position on the chromosome, which is as follows: *Mafa-A*, *-I* and *-B*, *-DRA*, -*DRB*, -*DQA1*, *-DQB1*, *-DPA1*, and *DPB1*, respectively (Watanabe et al. 2007). The exact order of loci within the duplicated gene regions, such as *Mafa-B* and -*DRB*, is unknown; hence, the listing of these alleles in the columns is arbitrary. The haplotypes are ordered vertically based on the *Mafa-A1* alleles and are numbered on an arbitrary basis. The list of 30 is extended by two more haplotypes that appear to be combinations of others. Number 4 is composed of haplotypes 3 and 21, whereas 22 may be the result of a crossing between 21 and 26. These recombined haplotypes are observed in four and two animals, respectively.

**Table 2:** All 32 MHC haplotypes present in the BPRC cynomolgus macaques. The gene regions are ordered in columns based on the position on the chromosome, with *Mafa-A1* on the telomeric side and *-DPB* on the centromeric side. The last columns contain numbers of haplotypes present in the BPRC cohort (*N* = 242) and the original colony (*N* = 390). The DRB alleles that are depicted in bold face are transcribed

	*Mafa-A1*	*Mafa-A2*	*Mafa-A4/5*	D6S2854	D6S2859	*Mafa-I*	*Mafa-B*	*Mafa-B*	*Mafa-B*	*Mafa-B*	
1	*001:01	*05:14		177, 196	201		*044:03	*056:01			
2	*003:01			177, 181, 196	171		*044:01:02				
3	*007:02		A4*14:02	181, 192	171	*01:09	*002:02	*046:07			
4	*007:02		A4*14:02	181, 192	171	*01:09	*002:02	*046:07			
5	*010:02	*05:17		196	169, 171		*033:03	*090:01			
6	*010:03	*05:06		181, 196	169, 171		*103:01	*104:04			
7	*018:05	*05:12:02	A4*14:01	173, 181, 196	171, 201		*002:01	*114:01			
8	*018:06	*05:34	A4*14	173, 199, 212	185, 201		*090:01				
9	*018	*05:16		173, 199, 216	185, 197		*137:03				
10	*031:01 a	*05:04		173, 181, 196	171, 199	*01:17	*051:02	*062:01	*065:01	*142:02	
11	*031:01 b	*05:04		173, 192, 196	171, 199	*03:01:01	*027:02	*147:01			
12	*031:02	*05:04		173, 181, 196	171, 199	*03:01:02	*027:01	*147:01			
13	*040:02			181, 221	196		*069:02				
14	*058:01			181, 212	185	*01:20	*007:03	*106:01			
15	*059:01	*05:08		181	199	*01:18	*109:01	*136:03			
16	*060:01			181, 192, 216	185		*036:01:02	*037:01	*045:01		
17	*061:01			192	145		*010:01				
18	*062:04			185, 192	null		*033:03	*090:01			
19	*063:01	*05:01		173, 192	169, 201		*046:01:01	*064:01	*104:01:01		
20	*063:02	*05:11		173, 192	169, 197	*01:10	*011:01	*075:01			
21	*064:01	*05:05		181, 192, 199	147, 201	*01:16	*025:01	*044:02			
22	*064:01	*05:05		181, 192, 199	147, 201		*044:01:01	*077:01			
23	*065:01:02	*05:02		169, 181, 196, 208	189, 199	*01:19	*021:01	*028:03	*068:02	*144:04	
24	*066:02	*05:10		181, 185, 212	185, 201	*01:15	*046:06	*095:02	*139:02		
25	*068:01	*05:15		173, 185, 196, 221	185, 197		*046:05	*064:02	*104:01:02		
26	*069:01			181, 192	169		*044:01:01	*077:01			
27	*070:01			181, 185, 196	171		*048:04	*102:01			
28	*071:01		A4*14	181, 196	173	*01:13:02	*013:04	*014:01	*034:02		
29	*071:03			173	169		*104:04	*162:01			
30	*072:01	A2*05	A5*30:01	173, 192	null		*069:01	*075:02			
31	*092:03		A5*30:02	192, 216	167		*148:01:02	*150:02			
32	*104:01			181, 189, 196			*013:07	*137:02			

	*Mafa-DRA*	*Mafa-DRB*	2878	*Mafa-DRB*	2878	*Mafa-DRB*	2878	*Mafa-DRB*	2878	*Mafa-DRB*	2878
1	*01:02:05	DRB1*04:03	201	DRB*W37:01	223						
2	*01:03:08	DRB*W3:03:01	251	DRB*W7:02	287	DRB6*01:13:01	218				
3	*01:02:01:01	DRB1*03:12:01	247	DRB*W25:02	185	DRB6*01:07	208				
4	*01:02:20	DRB1*04:01	189	DRB5*03:16	169	DRB*W3:03:01	247	DRB6*01:13:02	178?		
5	*01:02:01:01	DRB1*03:21	181	DRB1*10:10	199	DRB6*01:24	197				
6	*01:01:09	DRB1*07:04	191	DRB1*03:08:02	234	DRB*W6:05	281				
7	*01:03:01	DRB*W4:05	211	DRB*W25:04	209	DRB6*01:14	226				
8	nd	DRB1*04:01	189	DRB5*03:01:01	169	DRB4*01:01	257	DRB6*01:13:02			
9	nd	DRB1*03:12:01	249	DRB*W25:02	185	DRB6*01:07	208				
10	*01:03:01	DRB*W20:01	273	DRB*W66:01	195	DRB6*01:08	204				
11	*01:02:01:01	DRB1*04:01	189	DRB5*03:01:01	169	DRB4*01:01	253	DRB6*01:13:02	178		
12	*01:03:03	DRB*W68:01	209	DRB*W25:05	nd	DRB6*01:11	178				
13	*01:02:05	DRB1*03:09	209	DRB*W20:01	283	DRB6*01:07	214?				
14	*01:02:01:01	DRB1*07:04	199	DRB*W53:01	219	DRB5*03:05	173				
15	*01:03:01/03	DRB1*03:17	193	DRB*W6:07	203	DRB6*01:06	210	DRB6*01:12	192	DRB*W67:01	191
16	*01:03:01	DRB1*04:11	229	DRB*W36:04	229	DRB6*01:15	214				
17	*01:01:01	DRB1*04:01	187	DRB5*03:01:01	169	DRB4*01:02	229	DRB6*01:13:02	180?		
18	*01:02:01:01	DRB1*10:02	204	DRB*W49:01:02	265	DRB6*0109	184				
19	*02:01:01:01	DRB*W5:01	189	DRB*W21:01	227	DRB6*01:01	204				
20	*01:02:01:01	DRB1*10:02	208	DRB*W49:01:01	263	DRB6*01:09	184				
21	*01:02:20	DRB1*04:01	189	DRB5*03:16	169	DRB*W3:03:01	247	DRB6*01:13:02	178?		
22	*01:02:01:01	DRB1*03:06:01	193	DRB5*03:09	225	DRB*W65:01	227	DRB6*01:12	188		
23	*02:01:01:01	DRB*W5:01	221	DRB*W21:01	229	DRB6*01:01	206				
24	*01:02:21	DRB3*04:01	237	DRB5*03:06	169	DRB6*01:10	186				
25	*01:02:01:01	DRB1*03:13	209	DRB*W36:01	223	DRB6*01:05	208	DRB*W1:08	304?		
26	*01:02:01:01	DRB1*03:06:01	193	DRB5*03:09	225	DRB*W65:01	227	DRB6*01:12	188		
27	*01:03:02	DRB1*03:16	195	DRB*W40:01	203	nd	181				
28	*01:02:01:01	DRB1*10:02	218	DRB*W49:01:01	259?	DRB6*01:09	184				
29	*01:10:02	DRB4*01:03	223	DRB5*03:04	169						
30	*01:09	DRB*W20:02	220	DRB*W25:06	nd	DRB6*01:11	180				
31	*01:10:01	DRB1*03:08:01	222	DRB1*10:04	199	DRB6*01:09	183				
32	nd	DRB1*03:12:01	247	DRB*W25:02	185	DRB6*01:07	208				

	*Mafa-DQA1*	*Mafa-DQB1*	*Mafa-DPA1*	*Mafa-DPB1*	242	390					
1	*05:04	*17:05	*04:02	*03:04	5	6					
2	*24:02:03	*18:08	*07:04	*21:01	1	2					
3	*23:01	*18:04	*02:03	*04:01	29	41					
4	*23:01	*18:04	*02:03	*04:01	3	4					
5	*01:14	*06:23	*02:22	*15:08	5	9					
6	*05:07	*17:06:02	*02:21	*06:03	12	18					
7	*01:15	*06:15	*02:01	*15:03	11	18					
8	*01:07	*06:08	*02:16	*06:08	5	9					
9	*23:01	*18:04	*04:01	*02:05	9	14					
10	*26:01:02	nd	nd	*01:07	10	16					
11	*01:07	*06:08	*04:01	*03:03	4	6					
12	*23:01	*18:04	*04:01	*03:03	7	13					
13	*01:13	*06:17	*02:03	*04:01	5	12					
14	*05:02	*17:02:01	*07:02	*19:03	8	12					
15	*05:09	*17:04	*10:01	*18:01	17	27					
16	*05:03:02	*16:01	*02:08:01	*06:01	2	2					
17	*01:06	*06:11	*07:04	*21:01	6	8					
18	*05:03:01	*16:01	*04:01	*02:02	2	3					
19	*24:03	*18:01:01	*07:02	*19:03	12	18					
20	*05:03:01	*16:01	*02:02	*09:02	6	12					
21	*01:06	*06:11	*02:13:02	*15:02	23	34					
22	*24:06	*18:06	*04:01	*03:01	0	2					
23	*24:03	*18:01:01	*02:23	*06:08	6	8					
24	*23:01	*18:04	*02:20	*15:05	17	26					
25	*01:02	*06:07:01	*07:04	*20:01	7	13					
26	*24:06	*18:06	*04:01	*03:01	4	5					
27	*05:03:02	*16:01	*02:03	*04:01	11	20					
28	*05:08	*17:02:02	*02:19	*10:01	4	17					
29	*24:02:01	*18:08	*02:06:02	*07:01	0	1					
30	*05:03:01	*16:01	*02:06:02	*07:01	9	9					
31	*01:08:02	*06:01:02	*02:13:02	*15:02	2	4					
32	*01:15	*06:01:02	*04:01 ?	*02:05 ?	0	1					

The *Mafa-A*, *-B*, and *-I* alleles were detected by full-length sequencing on RNA, and thus, they represent only transcribed genes (Otting et al. 2009). The *Mafa-A*, *-B*, and *-I* alleles are unique for each haplotype, with a few exceptions. The combinations Mafa-A1*031:01/A2*05:04 and Mafa-B*033:03/B*090:01 are both observed in two haplotypes. Furthermore, two other alleles Mafa-B*104:04 and Mafa-B*147:01 are seen in two haplotypes, however, in combination with different other *Mafa-B* alleles. Two Mafa-A4*14 sequences, indicated by the lineage-number only, are not confirmed by three identical clones, and the exact full-length sequences are still unknown.

All haplotypes may contain an allele of the oligomorphic *Mafa-I* gene, a locus that was first described in the rhesus macaque (Urvater et al. 2000). However, in most cases, the criterion of three identical full-length clones to define an allele was not fulfilled. As mentioned previously, each *Mafa-A/-B/-I* combination is associated with a unique microsatellite length pattern. The sizes of the D6S2854 and D6S2859 repeats are depicted in base pairs in the columns next to the *Mafa-A1* columns in Table 2. The lengths are based on comparison to size standards in the capillary electrophoresis and may differ slightly from the actual length of the PCR products. However, the position of the repeats within the *Mafa-A* region, in relation to the different loci *Mafa-A1*, *-A2*, and so on, is unknown. In 2010, the *Mafa-B* sequences that were submitted to the Nonhuman Primate part of the IPD-MHC database underwent a drastic renaming, with the aim of adjusting the lineage numbers to *Mamu-B* alleles in the rhesus macaques. Hence, the *Mafa-B* allele-names in Table 2 are different from those listed in the original paper that was published in 2009 (Otting et al. 2009). The old and new designations are available at http://www.ebi.ac.uk/ipd/mhc/nhp/nomenclature.html.

In contrast to the class I sequences, the sharing of alleles among haplotypes is observed within the *DR* region. The DRA*01:02:01:01 allele, for example, is present in 10 haplotypes, whereas *01:09 and *01:02:21 are unique for haplotypes 30 and 24, respectively. The *DRA* alleles are detected by full-length sequencing on RNA (Doxiadis et al. 2011b). In contrast to the *DRA* locus, of which only one copy is present per haplotype, the *DRB* region in macaques is well known for its wide variety of region configurations (de Groot et al. 2004, 2008). Region configurations are groups of haplotypes that differ in the number and contents of *DRB* loci. Within this cohort of animals, the *DRB* region has been investigated thoroughly, by sequencing of exon 2 on genomic DNA as well as by full-length sequencing on RNA (Doxiadis et al. 2010, 2011b). The alleles that are transcribed, as pointed out by full-length sequencing on RNA, are depicted in boldface in Table 2. The number of *DRB* loci varies from 2 to 5, as is evident in the haplotypes 1 and 15, respectively. Five *DRB* combinations are shared by haplotypes, with one example present in 3, 9, and 32, and another in 8, 11, and 17. However, these shared *DRB* combinations may show differences in *DRA* alleles as well as in microsatellite lengths. The microsatellite D6S2878, used for *DRB*-typing, is located in intron 2 of *DRB* genes. In contrast to the STRs in the *Mafa-A* region, it was possible to link each *DRB* allele to a particular STR length, by sequencing exon 2/intron 2 combinations. For these reasons, each column with *DRB* alleles in Table 2 has an adjacent column with the associated STR lengths.

The *DP* and *DQ* sequences that were generated in this study are listed in the last columns of Table 2. Several animals were subjected to repeated analyses, but for unknown reasons, we failed to detect *DPA* and *DQB* alleles corresponding to haplotype 10. Furthermore, haplotype 32 is based on one animal only, in which the other chromosome contained haplotype 9. Sequencing analyses in this animal yielded only DPA1*04:01/DPB1*02:05. It is possible that this animal is homozygous for the *DP* region, but it cannot be excluded that the second alleles were missed due to primer inconsistencies. An investigation of related animals is not possible, since this animal has no offspring in the BPRC population. In contrast to the class I sequences, the detected *DQ* and *DP* alleles are not always haplotype specific. The combination DPA1*02:03/DPB1*04:01, for example, is observed in three haplotypes, whereas DQA1*23:01/DQB1*18:04 is present in four combinations (combined haplotypes excluded). Other combinations, such as DPA1*02:20/DPB1*15:05 and DQA1*01:02/DQB1*06:07:01, are haplotype specific. When *DQ* and *DP* alleles are taken together, however, each haplotype appears to contain a unique combination of *DQA/DQB/DPA/DPB* alleles.

**Fig. 1:**
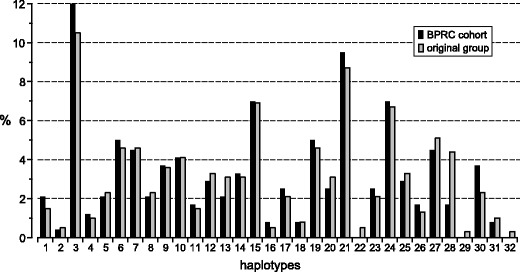
The haplotype distribution in the BPRC cohort and in the original macaque population at the University of Utrecht

As previously mentioned, the BPRC cohort is a subset of a colony that had been housed and pedigreed at the University of Utrecht for up to seven generations. In this cohort, 121 animals (242 haplotypes) are fully typed for MHC class I and class II markers. However, genomic DNA samples and lymphoblastoid B cells, as well as pedigree data pertaining to the original colony, were available. Hence, it was possible to extend the number of typed animals to 195 (390 haplotypes). In the last columns of Table 2, the haplotype distributions are presented, in absolute numbers. The percentages are depicted in Fig. 1. Comparison of these percentages shows that the cohort has not experienced a dramatic shift in the haplotype distribution. A reduction is observed for haplotype 28, with a percentage of 4.4 % in the original group and 1.7 % in the BPRC cohort. The other differences are all negligible. Three haplotypes, 22, 29, and 32, with low frequencies in the original population, are not present in the current cohort. Two animals are homozygous for their MHC; one of them has haplotype 3, the most common in the cohort, whereas the other is homozygous for haplotype 21.

## Concluding remarks

Here, we present cynomolgus macaques that are completely sequenced for the most significant MHC class I and class II genes, resulting in 32 distinct haplotypes. The estimated number of founding animals is around 30, which makes the number of haplotypes high in comparison to other cohorts (Mitchell et al. 2011). Additionally, the *Mafa-A* and *-DRB* regions are characterised by microsatellite typing. Using these high-throughput microsatellite techniques makes it easy to analyze the offspring of animals with these *Mafa-A* and *-DRB* markers and hence to deduce the accessory haplotype. Crossover events between *Mafa-A* and *-DRB* will be easily detected by using these techniques, and in these cases, further sequencing analyses of the particular infant can be performed. These techniques have already led to the definition of the two haplotypes 4 and 22, which may have been the result of recombination in recent generations. Similar observations were made in cynomolgus macaques of Mauritian origin (O’Connor et al. 2007). Crossover events on the centromeric side of the *DRB* region, however, are not detected with current microsatellite typing. Additional sequencing of DPB may be necessary to exclude these events, together with the investigation of polymorphic microsatellites on the centromeric side of the MHC region. The STR’s D6S2876, DS62747, D6S2745, and D6S2771, used by Mitchell and coworkers, may be appropriate candidates to type the class II region. These analyses may also be a starting point for comparison of our cohort to the Indonesian animals investigated by this research group (Mitchell et al. 2011).

This MHC-typed cohort of animals facilitates the choice of appropriate animals for research on immune-related diseases. Haplotypes 19 and 20, for example, are identical to the Mauritian haplotypes M1 and M3 (Budde et al. 2010), also known as H1 and H3 (Mee et al. 2009). These haplotypes are relevant to SIV pathogenesis and vaccine evaluation and are present in 12 and 4 individuals in the BPRC cohort, respectively.
